# An Automated Blood Pressure Display for Self-Measurement in Patients With Chronic Kidney Disease (iHealth Track): Device Validation Study

**DOI:** 10.2196/14702

**Published:** 2020-04-02

**Authors:** Victoria Mazoteras-Pardo, Ricardo Becerro-De-Bengoa-Vallejo, Marta Elena Losa-Iglesias, Daniel López-López, César Calvo-Lobo, David Rodríguez-Sanz, Eva María Martínez-Jiménez, Patricia Palomo-López

**Affiliations:** 1 Facultad de Enfermería, Fisioterapia y Podología Universidad de Complutense de Madrid Madrid Spain; 2 Faculty of Health Sciences Universidad Rey Juan Carlos Alcorcón Spain; 3 Research, Health and Podiatry Group Faculty of Nursing and Podiatry, Departament of Health Sciences Universidade da Coruña Ferrol Spain; 4 Facultad de Fisioterapia y Enfermería, Departamento de Enfermería Universidad Castilla la Mancha Toledo Spain; 5 University Center of Plasencia Universidad de Extremadura Plasencia Spain

**Keywords:** iHealth Track, validation, blood pressure, heart rate, International Protocol

## Abstract

**Background:**

Hypertension is a global public health issue and is closely related to chronic kidney disorder (CKD). In people with CKD, strict monitoring of blood pressure is an important part of therapy.

**Objective:**

The aim of this research was to validate the iHealth Track blood pressure monitoring device for patients with CKD according to the European Society of Hypertension International Protocol 2010 (ESH-IP2).

**Methods:**

In total, 33 patients who received hemodialysis in Plasencia participated in the study. There were 9 successive measurements made, which conformed to the ESH-IP2. We calculated the differences between the standard reference device (Omron M3 Intellisense) and the test device (iHealth Track) for blood pressure and heart rate values. For 99 total comparisons of paired measurements, we classified differences into various categories (≤5 mmHg, ≤10 mmHg, and ≤15 mmHg for blood pressure; ≤3, ≤5, and ≤8 beats per minute for heart rate).

**Results:**

In 90 of 99 systolic blood pressure and 89 of 99 diastolic blood pressure comparisons between the devices, measurement differences were within 5 mmHg. In 81 of 99 heart rate comparisons between the devices, measurement differences were within 3 beats per minute. The mean differences between the test and reference standard measurements were 3.27 (SD 2.99) mmHg for systolic blood pressure, 3.59 (SD 4.55) mmHg for diastolic blood pressure, and 2.18 (SD 2.75) beats per minute for heart rate. We also observed that for both systolic and diastolic blood pressure, 31 of 33 participants had at least two of three comparisons between the devices with measurement differences less than 5 mmHg. For heart rate, 28 of 33 patients had at least two of three comparisons between the devices with measurement differences less than 3 beats per minute.

**Conclusions:**

To our knowledge, this is the first study to show that iHealth Track meets the requirements of the ESH-IP2 in patients with CKD. Therefore, the iHealth Track is suitable for use in renal patients.

## Introduction

Chronic kidney disorder (CKD) is a syndrome defined by persistent alterations in renal function or structure that cause complications in a patient's health. Some of the structural anomalies may be tumors, cysts, malformations, or atrophies. In addition, renal dysfunction can be manifested through alterations in the output or grade of urine, increased risks of intellectual disabilities in children, edema, and hypertension [1,2].

In fact, the diseases most related with CKD are hypertension and diabetes, especially in high- and middle-income countries [3,4]. Hypertension may simply be a consequence of CKD [5,6] or both a cause and consequence of CKD [7,8]. Hypertension may be due to hypervolemia or activation of the renin-angiotensin system or neurohumoral (catecholamine and aldosterone) axis. In addition, sometimes high blood pressure (BP) originates from calcineurin or corticosteroid inhibitors used to treat underlying kidney disorders [9].

The interaction between CKD and hypertension is complex and increases the probability of cerebrovascular and cardiovascular problems [9-11]. In several studies, cardiovascular events and deaths from any cause were reduced when systolic BP was <120 mmHg (compared to <140 mmHg) in patients with CKD and hypertension but not diabetes [11-13]. Therefore, strict control of BP is important for CKD therapy [14].

Monitoring of BP should be done with devices that are easy to use and accurate [11,15-18]. These devices must be tested and validated by independent experts (eg, the British Hypertension Society [19], the Association for the Advancement of Medical Instrumentation [20], and the European Society of Hypertension [21,22]) with protocols validated and designed expressly for BP monitoring [23]. The purpose of this study was to validate the iHealth Track BP monitoring device for self-measurement in patients with CKD, according to the European Society of Hypertension International Protocol 2010 (ESH-IP2). Therefore, the hypothesis of this study was that iHealth Track would be valid for the self-measurement of BP and heart rate (HR) in renal patients according to the ESH-IP2.

## Methods

### Ethical Information

The study protocol was approved by the Institutional Local Research and Ethical Committee (Universidad de Extremadura, Badajoz, Spain; record number 152/2019). In conducting this study, we complied with the ethical principles of the Declaration of Helsinki [24], including any emendations between 2000 and 2013. All participants provided signed informed consent prior to participating in this study.

### The Devices

#### Omron M3 Intellisense

The standard device we used for reference was the Omron M3 Intellisense (Omron Healthcare, Kyoto, Japan), which has been validated according to the International Protocol for the general population [25] as well as CKD patients [26]. We purchased the Omron M3 Intellisense monitor from a local marketplace. The Omron M3 Intellisense is an oscillometric and automated upper-arm device for home BP monitoring. The device’s standard arm cuff is 22 to 32 cm around, and a large cuff is also available for arm circumferences of 32 to 42 cm. The device uses IntelliSense technology to produce comfortable, controlled inflation without the need for pressure presetting or reinflation.

#### iHealth Track

The test device was the iHealth Track automatic appliance with serial number KN-550BT (iHealthLabs Europe, Paris, France), which registers brachial BP with the oscillometric protocol. It detects BP between the range of 0 mmHg to 300 mmHg (measuring precision ±3 mm Hg) and HRs within the range of 40 to 180 beats per min (measurement precision ±5%). The device’s arm cuff is 22 to 42 cm around.

The device’s liquid crystal display screen shows the measured systolic (S) BP, diastolic (D) BP, and HR values. The device unit has enough memory for 99 recordings. Additionally, this device unit can be used with Apple Bluetooth 4.0 devices and certain Android Bluetooth 4.0 cellular phones through an application named Health MyVitals. This means that BP and HR data can be stored on wireless devices connected to iHealth Track and then displayed graphically.

### Patients and Recruitment

We recruited patients with CKD who attended the Fresenius Medical Care dialysis clinic in Plasencia, Spain. A total of 33 patients who met the selection criteria participated. The inclusion criteria were adults 25 years of age or older that received hemodialysis. We sought at least 10 male participants and 10 female participants. The exclusion criteria, which were created according to the ESH-IP2 [21,22], were sustained arrhythmia, circulatory problems where use of the cuff is contraindicated, and pregnancy.

### Research Protocol

The professional validation team consisted of 2 nurses with senior experience (more than 6 years) in BP measurement. The measurement area was correctly conditioned to a suitable temperature, and factors that could affect the records, such as noise or distractions, were removed [21,22]. All measurements were made in the same room. The color of the room was white.

After dialysis, each patient first reported information regarding their sex, age, height, and dry weight. In addition, we calculated participants’ BMI using Quetelet´s index in kg/m^2^. The circumference of the patient’s arm was measured to ensure that the cuff size was adequate.

Next, patients sat in the measurement room and BP measurements were started after a 10- to 15-minute rest period. Each patient was seated in a standard-size plastic chair with a backrest and armrests.

In total, 9 consecutive measurements were made on each participant with the Omron M3 Intellisense (5) and the iHealth Track (4) as follows [21,22]:

- (BPA): input BP, by the standard device unit
- (BPB): device BP detection by the test device unit
- (BP1): standard device unit
- (BP2): test device unit
- (BP3): standard device unit
- (BP4): test device unit
- (BP5): standard device unit
- (BP6): test device unit
- (BP7): standard device unit

During the measurements, participants remained calm, quiet, seated, and still. Participants kept their backs straight and feet on the floor in a parallel position (ie, without crossing their legs). They rested their arms on a flat surface with their palms facing upwards and their elbows slightly flexed so that their fists were at the height of their hearts.

BP records were made at heart level on the right arm in 31 participants and on the left arm in 2 participants (because of an arteriovenous fistula on the right arm). The standard cuff size (22-32 cm) for the Omron M3 Intellisense was used for all men (20). For women, the standard cuff (22-32 cm) was used for 11 participants, and the large cuff (32-42 cm) was used for 2 participants. Since the iHealth Track has only one cuff size (22-42 cm), all measurements were taken with it. The interval between one measurement and the next was 30 to 60 seconds [22]. 

All measurements were made for each participant during their hemodialysis appointment, after the dialysis was complete. The relative values were then used to calculate the mean difference between the reference device readings and the test device readings. All participants were receiving hemodialysis in the Fresenius Medical Care dialysis clinic in Plasencia.

### Data Analysis

We analyzed the data with the software SPSS Statistics, version 19.0 (IBM, Armonk, New York). We reported the findings as mean (SD).

According to the ESH-IP2, the accuracy of a device is based on a comparison between the test device (iHealth Track) and the standard reference device (Omron M3 Intellisense) measurements. For each participant, we first compared measurements BP2, BP4, and BP6 with measurements BP1, BP3, and BP5, respectively, and then compared measurements BP3, BP5, and BP7 with each other. The most favorable comparisons were used.

In our comparisons, we classified differences for both SBP and DBP, separately, by whether paired values were within 5, 10, or 15 mmHg [22], and whether paired values for HR were within 3, 5, or 8 beats per minute (BPM). We determined whether the test device passed the ESH-IP validation protocol. Part 1 of the validation process concerns the number of differences allowed in the specified ranges of each measure (SBP, DBP, and HR) for comparisons of individual measurements between devices (99 measurements) [22]. Part 2 concerns the comparisons between devices of each measure for individual participants (33) [22].

Moreover, we produced Bland-Altman plots [27,28] to display the agreement between the two devices (the iHealth Track and the Omron M3 Intellisense). These plots show the difference between each pair of measurements on the y-axis against the mean of each pair of measurements on the x-axis (for SBP, DBP, and HR).

## Results

### Participants

Of the 34 participants we recruited, 33 completed the study successfully (one was excluded for device failure). The 33 participants included 20 men and 13 women. Table 1 shows a summary of their biometric characteristics.

**Table 1:** Participants’ biometric characteristics.

Variables	Total sample (N=33)		Males (N=20)		Females (N=13)		
	Mean (SD)	Range	Mean (SD)	Range		Mean (SD)	Range
Age (years)	71.24 (11.76)	47.0-88.0	70.25 (11.42)	47.0-85.0		72.77 (12.57)	47.0-88.0
Weight (kg)	70.48 (15.87)	46.0-101.0	70.18 (12.95)	47-100.0		70.94 (20.16)	46.0-101.0
Height (cm)	162.24 (9.87)	141.0-180.0	167.10 (4.58)	160.0-180.0		154.77 (11.24)	141.0-174.0
BMI (kg/m^2^)	26.95 (6.72)	18.0-44.0	25.07 (4.08)	18.0-33.0		29.84 (8.90)	19.0-44.0
Arm circumference (mm)	265.0 (33.12)	220.0-350.0	264.75 (26.13)	220.0-320.0		265.0 (33.12)	220.0-350.0

### Blood Pressure Measurements

The iHealth Track BP device validation results were taken in accordance with the ESH-IP2. The mean differences between the reference standard and test devices were 3.27 (SD 2.99) mmHg for SBP and 3.59 (SD 5.28) mmHg for DBP. In 90 out of 99 SBP and 89 out of 99 DBP comparisons between the devices, measurement differences were within 5 mmHg, exceeding the ESH-IP thresholds (>72 comparisons for SBP and >64 comparisons for DBP). Additionally, in 95 out of 99 SBP and 94 out of 99 DBP comparisons between the devices, measurement differences were within 10 mmHg, also exceeding the ESH-IP thresholds (>86 comparisons for SBP and >80 comparisons for DBP). Moreover, in 98 out of 99 SBP and 94 out of 99 DBP comparisons between the devices, measurement differences were within 15 mmHg, which again surpasses the ESH-IP thresholds (>95 comparisons for SBP and >92 comparisons for DBP). Therefore, the iHealth Track passed part 1 of the validation protocol for BP monitoring.

Regarding part 2 of the ESH-IP2, 31 out of 33 participants had at least two of the three comparisons between devices with measurement differences within 5 mmHg for both SBP and DBP, exceeding the ESH-IP threshold (>23 participants). One participant had all three comparisons for both SBP and DBP with measurement differences greater than 5 mmHg, which is less than the ESH-IP maximum of 3 participants. Given these results, the iHealth Track also passed part 2 of the validation protocol for BP. Thus, because the iHealth Track passed parts 1 and 2 of the BP validation protocol, it passed part 3 of the protocol, overall validation.

### Heart Rate Measurements

The validation findings for the iHealth Track HR monitoring device were taken according to the ESH-IP2. The mean difference between the reference standard and test devices was 2.18 BPM (SD 2.75). In comparisons between devices, 81 out of 99 pairs of measurements were within 3 BPM, 91 out of 99 were within 5 BPM, and 96 out of 99 were within 8 BPM. These results indicate that the iHealth Track passed part 1 of the validation protocol for HR.

Regarding part 2 of the ESH-IP2, 28 out of 33 patients had at least two of three comparisons between devices with measurement differences within 3 BPM, which exceeds the ESH-IP threshold (>23 participants). Only 2 participants had all three HR comparisons with measurement differences greater than 3 BPM, which is less than the ESH-IP maximum of 3 participants. Therefore, the iHealth Track passed part 2 of the HR validation protocol and, consequently, part 3 (overall validation) as well.

**Figure 1:**
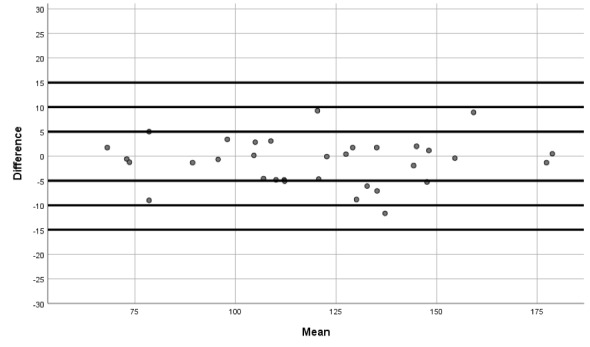
Bland-Altman graph of systolic blood pressure differences between the iHealth Track and the Omron M3.

**Figure 2:**
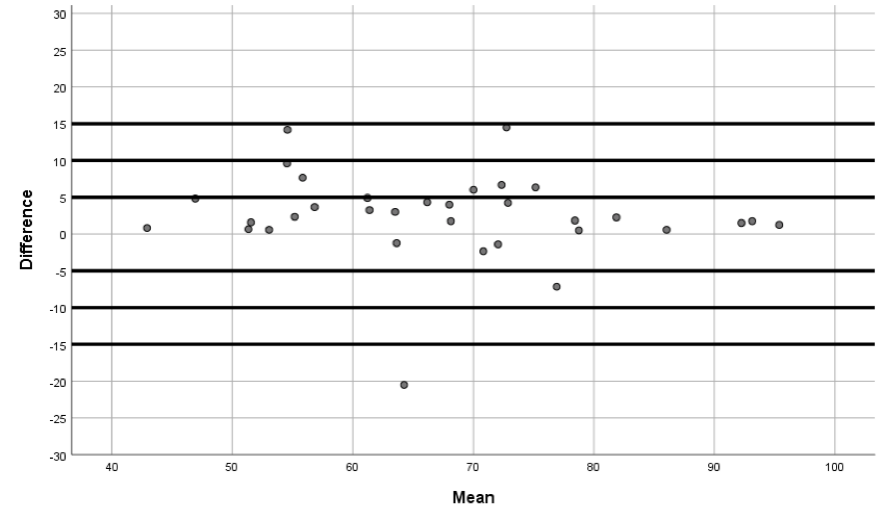
Bland-Altman graph for diastolic blood pressure differences between the iHealth Track and the Omron M3.

**Figure 3:**
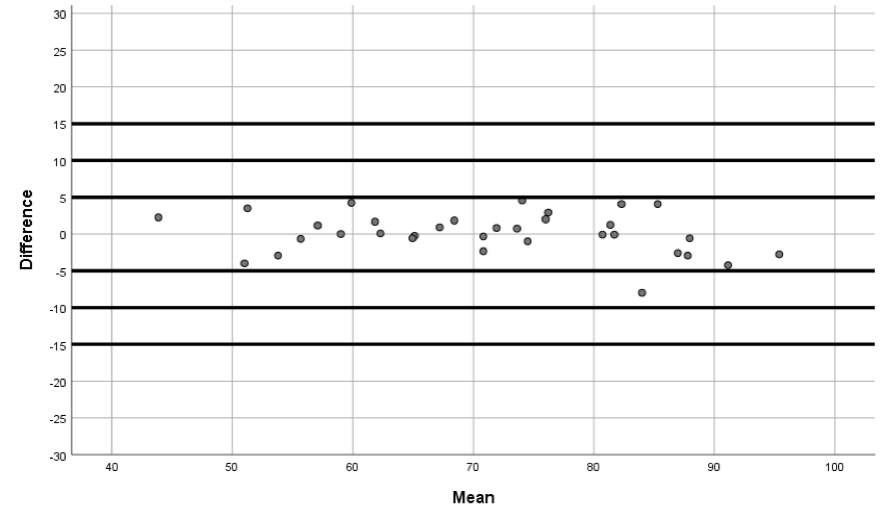
Bland-Altman graph for heart rate differences between the iHealth Track and the Omron M3.

The Bland-Altman graphs (Figures 1-3) give further information on the performance of the iHealth Track device. The graphs show that the measurement differences between the devices were fairly constant across the ranges of SBP, DBP, and HR.

## Discussion

This study is the first to validate the accuracy of the iHealth Track device for BP and HR recordings in patients with CKD. The results indicate that the iHealth Track, as used in renal patients, passed the ESH-IP2 validation requirements. We previously validated the iHealth Track device for the general population following ESH-IP2 [29].

This study showed two limitations. Although the iHealth Track has been validated in the general population and now in patients with CKD, we cannot necessarily extrapolate our results to other specific populations. In addition, patients with CKD have stiffer arteries than other people [9,11,14]. We did not investigate arterial stiffness, but it would be useful to assess it in future validation studies.

The ESH-IP2 for home BP monitoring highlights the need for specific validation in patients with end-stage renal disease [14,30] as strict control of hypertension is required in these patients [9,14].

However, there are few devices that have been validated in patients with renal disease [26,31-34]. Akpolat et al [26] validated Omron M3 HEM-7051 in patients with CKD according to the ESH-IP2 revision. They used the mercury sphygmomanometer as their standard reference device and included 66 participants, rather than 33. The results were similar to ours, since both studies passed the ESH-IP revision’s two phases of validation. However, the number of differences included in the category of 5 mmHg according to the ESH-IP2 were better for the iHealth Track for both SBP and DBP (ie, iHealth Track achieved higher differences than Omron). The differences obtained with the HRs cannot be contrasted as HR was not measured by Akpolat et al. Likewise, our findings cannot be compared with the rest of the validation studies found [31-34], since none followed the ESH-IP2 validation requirements. We believe that more validation studies for BP monitoring devices are necessary for patients with CKD.

The purpose of the ESH-IP2 was to simplify the previous protocols of the British Hypertension Society (BHS) [19] and the Association for the Advancement of Medical Instrumentation (AAMI) [20]. However, the protocol does have some shortcomings. First, the major limitation is that it is underpowered with only 33 participants (99 measures) required rather than the 85 participants (255 measures) required by the previous AAMI and BHS validation protocols [19,20]. Second, the ESH-IP2 does not indicate the number of validation studies needed to establish the accuracy of a device. According to some experts, at least two validation studies should be performed in different centers and in different populations before accepting the device as accurate [35]. Therefore, it is valuable to evaluate BP devices in diverse specific populations before they are used widely in clinics and homes. Third, the ESH-IP2 imposes certain gender requirements and limits validation studies to individuals older than 25 years who have BPs within specific ranges. Therefore, device accuracy remains unknown in children, adolescents, young adults, and patients with extreme BP values. Finally, the ESH-IP2 does not mention explicit criteria for validation in specific populations. Following the start of our study, in March 2019, the AAMI/ESH/ISO Universal Standard was published as the recommended standard for validation of BP measuring devices. [36]. This standard includes criteria for the validation of BP devices in specific populations. This will be considered in our future validations.

Our study is the first to show that the iHealth Track device meets the requirements of ESH-IP2 in patients with CKD. Future versions of the ESH-IP should include explicit criteria for validation in specific populations. Validation of this device would be valuable in other specific populations such as pregnant women, older adults, and patients with arrhythmias.

## Abbreviations

AAMI: Association for the Advancement of Medical Instrumentation
BHS: British Hypertension Society
BP: blood pressure
BPM: beats per minute
CKD: chronic kidney disorder
D: diastolic
ESH-IP2: Hypertension International in European Society protocol 2010
HR: heart rate
ISO: International Organization for Standardization
S: systolic
